# Parents' Experiences Discussing Pediatric Vaccination with Healthcare Providers: A Survey of Canadian Naturopathic Patients

**DOI:** 10.1371/journal.pone.0022737

**Published:** 2011-08-02

**Authors:** Jason W. Busse, Rishma Walji, Kumanan Wilson

**Affiliations:** 1 Institute for Work & Health, Toronto, Ontario, Canada; 2 Department of Clinical Epidemiology and Biostatistics, McMaster University, Hamilton, Ontario, Canada; 3 Department of Health and Aging and Society, McMaster University, Hamilton, Ontario, Canada; 4 Canadian College of Naturopathic Medicine, Toronto, Ontario; 5 The Ottawa Health Research Institute, University of Ottawa, Ottawa, Ontario, Canada; Children's Hospital of Eastern Ontario, Canada

## Abstract

**Background:**

Parents who choose to selectively vaccinate or avoid vaccination for their children may do so at risk of compromising relations with their family physician or pediatrician. Groups that are associated with reduced rates of pedicatic vaccination, such as parents who access naturopathic care, may be particularly vulnerable to this issue.

**Methodology/Principal Findings:**

In March through September 2010, we administered a 26-item cross-sectional survey to 129 adult patients, all of whom were parents with children ≤16 years of age, presenting for naturopathic care in Ontario, Canada. Ninety-five parents completed the survey (response rate 74%), and only 50.5% (48 of 95) reported that their children had received all recommended vaccines. Most parents (50.5%; 48 of 95) reported feeling pressure to vaccinate from their allopathic physician and, of those who discussed vaccination with their physician, 25.9% (21 of 81) were less comfortable continuing care as a result. Five percent (4 of 81) of respondents were advised by their physician that their children would be refused care if they decided against vaccination. In our adjusted generalized linear model, feeling pressure to vaccinate (odds ratio [OR] = 3.07; 95% confidence interval [CI] = 1.14 to 8.26) or endorsing a naturopathic physician as their most trusted source of information regarding vaccination (OR = 3.57; 95% CI = 1.22 to 10.44) were associated with greater odds of having a partially vaccinated or unvaccinated child. The majority (69.6%; 32 of 46) of parent's with partially vaccinated or unvaccinated children reported a willingness to re-consider this decision.

**Conclusions/Significance:**

Use of naturopathic care should be explored among parents in order to identify this high-risk group and engage them in discussion regarding pediatric vaccination to encourage evidence-based, shared decision making. Physicians should ensure that discussions regarding vaccination are respectful, even if parents are determined not to vaccinate their children.

## Introduction

Pediatric vaccination is one of the most successful public health interventions for reducing infant morbidity and mortality. Because of the demonstrated importance of vaccination to protect children from disease, many physicians strongly advocate that children be vaccinated. However, this advocacy on behalf of the child can sometimes bring them into conflict with parents in a manner that can be perceived as confrontational [1]. In 2002 Flanagan-Klygis et al. surveyed a random sample of 1004 American pediatricians (302 surveys were used for analysis) and found that, hypothetically, 39% (115 of 295) would dismiss a family for refusing all vaccinations and 28% (81 of 295) would dismiss a family for refusing select vaccinations [2]. The potential for parent-physician conflict over vaccination can be counterproductive and may have adverse implications for the long-term healthcare of children.

Naturopathic medicine is a popular complementary and alternative medicine (CAM) therapy that incorporates a range of modalities such as dietary and lifestyle counselling, homeopathic medicine, massage, acupuncture, and joint manipulation, with an emphasis on supporting health rather than combating disease [3], [4]. In 2003 there were 642 practicing members of the Canadian Naturopathic Association and by 2011 this number had more than doubled to 1313 members [5], [6]. The 2007 National Health Interview Survey found that approximately 729,000 U.S. adults and 237,000 children had received naturopathic care in the previous year [7], and a survey of randomly sampled CAM providers practicing in four American states found that children and adolescents comprised more than 10% of all visits to naturopathic physicians, compared to only 1% to 4% for other CAM providers [8].

Parents who seek naturopathic care may experience greater conflict with their pediatrician or family physician regarding the decision to vaccinate their children. Vaccination appears to be a contentious issue for some naturopathic doctors and students [9]–[11], and our review of 482 pediatric and adolescent charts of patients who presented to the Canadian College of Naturopathic Medicine (CCNM) found that, among the 316 charts that recorded vaccination status, 4.4% reported partial vaccination status and 8.9% reported being unvaccinated [5] which was below national immunization rates (e.g. the 2002 National Immunization Coverage Survey found that only 6% of Canadian children were not immunized against measles, mumps and rubella by age 2) [12].

Understanding the experiences of naturopathic patients regarding the decision to vaccinate their children may provide opportunities to improve doctor-patient discussions on this topic and increase pediatric vaccination rates among this potentially vulnerable population. We sought to explore the nature of discussions regarding pediatric vaccination that parents who seek naturopathic care have had with their healthcare providers, the vaccination status of their children, and if discussing vaccination had affected doctor-patient relationships.

## Methods

### Ethics Statement

Approval for our survey was granted by the CCNM ethics review board. Participants received a disclosure letter detailing the intent of the survey and explicit instructions that they could choose not to complete the survey. Informed consent was obtained verbally from all participants in order to facilitate administration of our survey, and this procedure was approved by the CCNM ethics review board.

### Questionnaire Development

With the assistance of epidemiologists and content experts, and reference to the previous literature [13], we developed a 26-item, English language questionnaire to examine the experiences of parents under naturopathic care regarding their discussions of pediatric vaccination with healthcare providers and the vaccination status of their children. The final questionnaire was comprised of closed-ended questions as a previous report has shown that open-ended formats are associated with a higher risk of incomplete questionnaires [14]. We also included an option for respondents to provide written comments regarding any other thoughts they may have on vaccination or their interactions with healthcare providers in regards to vaccination. We pilot tested the final questionnaire with two naturopathic patients who were parents.

### Questionnaire Administration

In March through September 2010, parents with at least 1 child ≤16 years of age presenting to either 1 of 3 naturopathic physicians practices at the CCNM clinic or 1 of 6 private naturopathic clinics in Ontario, Canada, were asked to complete our 26-item survey. Patients were informed that the purpose was to collect data on basic demographics, their child's/children's vaccination status, and discussions they had with their healthcare providers regarding pediatric vaccination. For those who consented, the survey was administered on presentation to the clinic and collected immediately. We selected parents attending naturopathic physicians as our previous research suggested an association with higher than average levels of partially vaccinated or unvaccinated children [5].

### Statistical Analysis

We generated frequencies for all collected data. Two of us (JWB, RW) grouped written comments, independently and in duplicate, according to themes to facilitate presentation. Disagreement was resolved by discussion. The responses were analyzed using thematic analysis [15]–[17]. The coding involved assigning unique labels to text responses that contained references to specific categories of information [18]. The codes corresponded to each belief conveyed by the responses.

We hypothesized, *a priori*, that the following variables may be associated with a higher likelihood of respondent's having at least 1 partially vaccinated or unvaccinated child: (1) if they reported feeling pressured to vaccinate their children; (2) if they reported lacking sufficient information to make an informed decision regarding vaccination; (3) if they reported discussing vaccination with their naturopathic physician; and (4) if they endorsed their naturopathic physician as their most trusted resource for information on vaccination. These 4 independent variables were entered into a multivariable logistic regression model. We calculated that we would require 40 completed surveys in which parents reported that at least 1 of their children was partially vaccinated or unvaccinated in order to ensure that our regression model was reliable (10 events for each independent variable considered) [19].

All comparisons were 2-tailed and a variable was considered statistically significant if it had a p-value<0.05 in the final multivariable model. We report the odds ratio (OR) and 95% confidence interval (CI) for each variable in the analysis. Goodness of fit for the multivariable regression model was determined by the Hosmer-Lemeshow (H-L) test. The H-L test measures predictive reliability by comparing the expected with the actual results of the dependent variable. The H-L is distributed approximately as χ^2^ with 8 degrees of freedom. Values of H-L less than 15.5 indicate a statistically good fit at the 0.05 level of significance [20]. We performed all analyses using IBM SPSS Statistics 18.0 statistical software (IBM Corporation, Somers, NY).

## Results

### Characteristics of Respondents

Of 129 eligible patients, 95 agreed to participate in our survey and provided a completed questionnaire (response rate of 74%); 69 from the CCNM clinic and 26 from private naturopathic clinics. Respondents were predominantly well-educated females at an average age of 36.7 years (SD = 6.1) and with a median of 2 children. The mean age of respondent's children was 7.8 (standard deviation = 5.9). Only half of respondents indicated that all of their children were fully vaccinated, and almost 1 in 4 parents advised they had at least 1 child who had not received any vaccines (Table 1). Of the 45 respondents with more than 1 child, 13 reported variations in vaccination status, which in all but 1 case entailed reduced vaccination status for their younger child/children; 5 parents reported full vaccination for their older and partial for their younger child/children, 4 parents reported fully vaccinating their older and not vaccinating their younger child/children, and 1 parent reported partially vaccinating their older child and not vaccinating their younger child. One parent reported not vaccinating their older child and partially vaccinating their younger child. Of the 47 parents who had one or more partially or unvaccinated child, the majority (69.6%; 32 of 46) reported that they would be prepared to re-consider this decision, 13.0% (6 of 46) would not reconsider, 17.4% (8 of 46) were unsure, and 1 respondent did not answer this question.

**Table 1:** Characteristics of Survey Respondents (n = 95).

**Female respondents, no. (%)**	81 (85.3)
**Age in years, mean (SD)**	36.7 (6.1)
**Education level**	
high school graduate, no. (%)	9 (9.5)
college graduate, no. (%)	26 (27.4)
university graduate, no. (%)	60 (63.2)
**No. of children, median (range)**	2 (1 to 7)
**Vaccination status of their children**	
all children fully vaccinated, no. (%)	48 (50.5)
≥1 child partially vaccinated, no. (%)	25 (26.3)
≥1 child unvaccinated, no. (%)	22 (23.2)
**Felt pressured to vaccinate their children, no. (%)** *	57 (60.0)
by their physician, no. (%)‡	48 (84.2)
by family, no. (%)‡	15 (26.3)
by their spouse, no. (%)‡	4 (7.0)
by friends, no. (%)‡	12 (21.1)
**Felt they had sufficient information to make an informed decision on vaccination, no. (%)**	53 (55.8)
**Whom do you most trust to provide good information on vaccination**	
family physician or pediatrician	15 (15.8)
naturopathic physician	30 (31.6)
both my family physician or pediatrician and naturopathic physician equally	43 (45.3)
unsure	7 (7.4)

Key: SD = standard deviation.

* = respondents could endorse more than 1 option.

‡ = respondents are limited to those parents that reported having felt pressure to vaccinate their children (n = 57).

The majority of parents (60.0%; 57 of 95) reported having been pressured to vaccinate their children, in most cases by their family physician or pediatrician. Only slightly more than half of respondents (55.8%; 53 of 95) endorsed that they had sufficient information to make an informed decision regarding whether to vaccinate their child/children. Many respondents (45.3%; 43 of 95) advised that they discussed vaccination with both their allopathic and naturopathic physicians; however, almost a third of respondents (31.6%; 30 of 95) indicated that they regarded their naturopathic physician as the most trustworthy resource for information on vaccination and only 15.8% (15 of 95) regarded their family physician or pediatrician as their most important source of information (Table 1).

### Discussing Pediatric Vaccination with their Allopathic Physician

The large majority (85.3%; 81 of 95) of respondents had discussed vaccination with their family physician or pediatrician (Table 2), and 85.2% (69 of 81) endorsed the belief that their physician held positive views towards vaccination. Forty-one percent (33 of 81) of parents viewed these discussions as positive, but 23.5% (19 of 81) did not. Forty-two percent (34 of 81) of parents reported that their discussions left them more comfortable choosing to vaccinate their child/children, whereas 17.3% (14 of 81) were less comfortable vaccinating and 25.9% (21 of 81) were less comfortable continuing care with their physician after their discussion. Respondents were evenly split as to whether information regarding vaccination from their physician was impartial, and 17.3% (14 of 81) endorsed that their discussions had introduced conflict into the doctor-patient relationship. Five percent of patients (4 of 81) noted that their physician had refused to provide future care to their child if they were not vaccinated, and 11.1% (9 of 81) were unsure if future care was dependent on their child's/children's vaccination status. In their written comments, another 2 respondents indicated they had left their physician's practice and 2 advised they had become hesitant to contact their physician due to conflict over vaccination. Twenty-eight percent of parents (23 of 81) advised that discussions regarding vaccination with their family physician or pediatrician influenced their decision to seek naturopathic care.

**Table 2:** Discussion of Vaccination with a Family Physician or Pediatrician (n = 81).

**Perception of physician's attitude towards vaccination**	
positive, no. (%)	69 (85.2)
neutral, no. (%)	11 (13.6)
negative, no. (%)	1 (1.2)
**Respondent's characterization of discussion**	
positive, no. (%)	33 (40.7)
neutral, no. (%)	28 (37.6)
negative, no. (%)	19 (23.5)
**Effect of discussion on decision to vaccinate children**	
more comfortable choosing to vaccinate, no. (%)	34 (42.0)
no impact, no. (%)	33 (40.7)
less comfortable choosing to vaccinate, no. (%)	14 (17.3)
**Effect of discussion on willingness to continue care with physician**	
more comfortable, no. (%)	28 (34.6)
no impact, no. (%)	32 (39.5)
less comfortable, no. (%)	21 (25.9)

	yes	no	unsure
**Was the information provided by your physician fair and impartial, no. (%)**	36 (44.4)	35 (43.2)	10 (12.3)
**Did your discussions introduce conflict into your relationship, no. (%)**	14 (17.3)	59 (72.8)	8 (9.9)
**Did your physician refuse future care to your child if they were not vaccinated, no. (%)**	4 (4.9)	68 (84.0)	9 (11.1)
**Did your discussions influence your decision to seek naturopathic care, no. (%)**	23 (28.4)	57 (70.4)	1 (1.2)

### Discussing Pediatric Vaccination with their Naturopathic Physician

Almost half of respondents (47.4%; 45 of 95) had discussed vaccination with their naturopathic physician (Table 3). Only 4.4% of parents (2 of 45) characterized the nature of these discussions as negative, and 62.2% (28 of 45) indicated that their naturopathic doctor held neutral views towards vaccination – defined as not strongly in favor of, or against, pediatric vaccination. The majority (62.2%; 28 of 45) advised that discussing vaccination with their naturopathic physician had no impact on their decision to vaccinate their child/children; however, 24.4% (11 of 45) reported that they were less comfortable with vaccinating after their discussion. Most parents (66.7%; 30 of 45) endorsed that their discussions made them more comfortable continuing with naturopathic care, and 84.4% (38 of 45) endorsed their naturopathic doctor's information regarding vaccination as fair and impartial.

**Table 3:** Discussion of Vaccination with a Naturopathic Physician (n = 45).

**Respondent's characterization of discussion**	
positive, no. (%)	29 (64.4)
neutral, no. (%)	14 (31.1)
negative, no. (%)	2 (4.4)
**Perception of naturopathic physician's attitude towards vaccination**	
positive, no. (%)	2 (4.4)
neutral, no. (%)	28 (62.2)
negative, no. (%)	15 (33.3)
**Effect of discussion on decision to vaccinate children**	
more comfortable choosing to vaccinate, no. (%)	6 (13.3)
no impact, no. (%)	28 (62.2)
less comfortable choosing to vaccinate, no. (%)	11 (24.4)
**Effect of discussion on willingness to continue care with naturopathic physician**	
more comfortable, no. (%)	30 (66.7)
no impact, no. (%)	14 (31.1)
less comfortable, no. (%)	1 (2.2)

	yes	no	unsure
**Was the information provided by your naturopathic physician fair and impartial, no. (%)**	38 (84.4)	2 (4.4)	5 (11.1)

### Factors Associated with Partially Vaccinated or Unvaccinated Children

Our univariable logistic regression model revealed 3 factors that were significantly associated with parent's having at least 1 child who was partially vaccinated or unvaccinated (Table 4). All 4 variables were entered into a multivariable regression model. The H-L test was not significant (χ^2^ = 11.15; p = 0.19), indicating goodness of fit for the multivariable regression model. In this adjusted analysis only feeling pressured to vaccinate (OR = 3.07; 95% CI = 1.14 to 8.26) and endorsing their naturopathic physician as their most trusted resource for information on vaccination (OR = 3.57; 95% CI = 1.22 to 10.44) remained significant (Table 4).

**Table 4:** Variables Associated with Naturopathic Patients having a Partially Vaccinated or Unvaccinated Child.

Variable	Univariable Analysis OR, 95% CI	p-value	Multivariable Analysis OR, 95% CI	p-value
**Feeling pressured to vaccinate**	4.21 (1.74 to 10.18)	<0.01	3.07 (1.14 to 8.26)	0.03
**Reporting a lack of sufficient information to make an informed decision on vaccination**	0.81 (0.36 to 1.83)	0.61	1.09 (0.43 to 2.80)	0.85
**Discussing vaccination with their naturopathic physician**	3.02 (1.30 to 7.03)	0.01	1.56 (0.59 to 4.13)	0.37
**Reporting their naturopathic physician as their most trusted resource for information on vaccination**	5.61 (2.10 to 15.03)	<0.01	3.57 (1.22 to 10.44)	0.02

OR = Odds Ratio.

95% CI = 95% Confidence Interval.

### Written Comments

Fifty-five percent (52 of 95) of respondents provided written comments which we grouped into three main themes: a need for more information on vaccination (36 comments), vaccine safety and efficacy (19 comments), and the effect of refusing to vaccinate, or selectively vaccinating, their child/children on healthcare provider relationships (18 comments). A number of respondents emphasized that good, unbiased resources regarding vaccinations are needed. Generally, physicians were seen to be biased sources of information by providing only pro-vaccine or incomplete information. For example:

“*Our child's pediatrician provided very little information on vaccines. She had a very biased and vague handout which seemed to be fuelled by a public health perspective (pro-vaccine)… I still feel ill equipped to make this decision.*”

Parents were often concerned with vaccine safety, particularly if their child, or someone they knew, had experienced a perceived vaccine-related adverse event. While some responses articulated a belief in efficacy and importance of vaccinations, many were concerned about potential risks:

“*I have collected a lot of info on vaccinations from many sources. After reading the info, I am quite scared to vaccinate my child. I… do not want my son's immune system to become compromised in any way!!*”

The third theme that emerged strongly from the data was how parent's choice regarding vaccination for their child affected relations with their health care provider. As a result, some parents chose to seek care elsewhere or were refused ongoing care by their physician. In other cases, if the parents perceived the relationship with their physician to be strained due to vaccination choices, they avoided appointments for fear of conflict:

“*I was met with stiff resistance from our pediatrician when I asked for more information before vaccinating my child… Because of our conflict, I was forced to find another doctor.*”

“*It was very difficult to find a doctor willing to take my son as a patient if I was not going to vaccinate. Many receptionists asked upon the first phone call and immediately told me they would not see my son if he was not vaccinated.*”

“*My experience with my family Dr. was if we didn't continue to vaccinate our child then we were making the wrong decision, which made me very uncomfortable. I have since hesitated before calling or discussing anything with her.*”

## Discussion

Our survey of parents attending naturopathic care found high rates of partial or unvaccinated status among their children, with only 50% of respondents having pursued all recommended pediatric vaccines. Most parents reported feeling pressure to vaccinate, primarily from their allopathic physician; 17.3% advised that discussions regarding vaccination with their family physician or pediatrician had introduced conflict into their relationship and 25.9% were less comfortable continuing care with their physician. Five percent of respondents were advised by their physician that their children would be refused care if they decided against vaccination.

Many respondents (44.2%) did not feel sufficiently informed to decide whether or not to vaccinate their children, and respondents most commonly endorsed both their allopathic and naturopathic physicians as trusted resources for information regarding vaccination. Allopathic physicians were largely seen as providing pro-vaccination material whereas discussion regarding vaccination with naturopathic physicians was seen as more balanced. Discussing vaccination with their allopathic physician influenced 28.3% of respondents to seek naturopathic care.

In our adjusted generalized linear model, feeling pressure to vaccinate or endorsing a naturopathic physician as their most trusted source of information regarding vaccination were both associated with threefold greater odds of having a partially vaccinated or unvaccinated child. Due to the cross-sectional design of our study we cannot establish if these associations are causal. For example, it may be that parent's who seek naturopathic care are more likely to reject vaccination for their children. The majority of parent's with partially or unvaccinated children (69.8%) reported a willingness to reconsider this decision.

As far as we are aware, ours is the first study to explore the association between parent's discussions with their healthcare providers regarding pediatric vaccination and the vaccination status of their children. Our high response rate, prospective design, and consecutive sample among a population with high rates of partially vaccinated or unvaccinated children strengthen our findings. There are some important limitations to this study. Our data are limited to self-report and responses were not confirmed. Our sample population was taken from a large Canadian naturopathic academic center and 6 private naturopathic clinics in Ontario, Canada, and our results may not be generalisable to other populations accessing naturopathic care.

A recent survey of 1004 American pediatricians (30.1% of surveys analyzed) suggested that approximately one third of pediatricians would discharge children from their practice if parents refused some or all pediatric vaccinations [2]. As far as we are aware, it is not illegal for physicians to deny future care to children on the basis of parent's refusal to vaccinate; however, the College of Physicians and Surgeons of Ontario does advise that refusal to treat patients may be grounds for a complaint of professional misconduct [21].

Our survey suggests that, among Canadian parents under naturopathic care, 5% were advised their children would be refused care if they opted not to pursue full vaccination. Another 2% felt compelled to leave their physician's practice and 1 in 4 parents felt less comfortable seeking care for their children as a result of discussion regarding vaccination. This suggests that allopathic physicians are less likely to discharge partially or unvaccinated children from their actual practice than when confronted with a theoretical scenario. However, there are a number of important services that physicians managing pediatric populations provide and it seems ill-advised to compromise this role based solely on parent's decisions regarding vaccination [22]. Our survey also suggests that current discussions with allopathic physicians regarding pediatric vaccination could be further optimized as many parents reported excessive pressure to vaccinate and felt that discussions were typically not balanced.

Parents who attend CAM providers, including naturopathic physicians, may have a greater risk of exposure to anti-vaccination arguments [10], [23]. Such arguments typically fall into 1 of 2 categories: vaccines are not effective and the risks of vaccination outweigh the benefits [24], [25]. No vaccine is 100% safe or 100% effective, and this is true of any health care intervention. However, opponents of vaccination frequently emphasize or exaggerate the adverse effects of vaccines, but fail to consider the consequences of compromised vaccination programs [24], [26]. Furthermore, although it is true that a number of published studies have implicated vaccines in certain disorders, these have generally not held up under investigative scrutiny. For example, an oft quoted 1998 study of 12 children by Wakefield et al. suggested a link between MMR vaccination and the development of autism [27].What antivaccinationists may fail to note is that larger trials failed to confirm these findings [28]–[30], and that Wakefield was subsequently found to have falsified his data [31] leading the Lancet to retract his publication in 2010 [32]. Parents who attend naturopathic care are more likely to avoid vaccinating or selectively vaccinate their children; however, most of our respondents advised that they would be willing to reconsider vaccinating their children. Use of naturopathic care should be explored among parents in order to identify this high-risk group and engage them in sufficient discussion regarding pediatric vaccination to address their concerns and encourage evidence-based, shared decision making. Physicians should ensure that discussions regarding vaccination are respectful, even if parents are determined not to vaccinate their children. Furthermore, allopathic physicians should look for opportunities to develop open lines of communication with naturopathic physicians involved in their patient's care to improve pediatric vaccination rates in this population.
